# The versatile reverse flow sural artery neurocutaneous flap: A case series and review of literature

**DOI:** 10.1186/1749-799X-3-15

**Published:** 2008-04-18

**Authors:** Syed Kamran Ahmed, Boris Kwok Keung Fung, Wing Yuk Ip, Margaret Fok, Shew Ping Chow

**Affiliations:** 1 Division of Hand and Foot, Department of Orthopaedics and Traumatology, Queen Mary Hospital, University of Hong Kong, Pok Fu Lam Road, Hong Kong, China

## Abstract

**Background:**

Reverse flow sural neurocutaneous flap has been utilized more frequently during the past decade to cover vital structures around the foot and ankle area. The potential advantages are the relatively constant blood supply, ease of elevation and preservation of major vascular trunks in the leg. The potential disadvantages remain venous congestion, donor site morbidity and lack of sensation.

**Methods:**

This descriptive case series was conducted at Queen Mary Hospital, Hong Kong, from 1997 to 2003. Ten patients having undergone reverse flow sural neurocutaneous flap were identified through medical records. There were six females (60%) and four males (40%), with an average age of 59.8 years. The defects occurred as a result of trauma in five patients (50%), diabetic ulcers in four (40%) and decubitus ulcer in one (10%) paraplegic patient. The defect site included non weight bearing heel in four (40%), tendo Achilles in two (20%), distal tibia in two (20%), lateral malleolus in one (10%) and medial aspect of the midfoot in one patient (10%). The maximum flap size harvested was 14 × 6 cm. Preoperative doppler evaluation was performed in all patients to identify perforators and modified plaster of paris boot was used in the post operative period. A detailed questionnaire was developed addressing variables of interest.

**Results:**

There was no flap failure. Venous congestion was encountered in one case. The donor site was relatively unsightly but acceptable to all patients. The loss of sensation in the sural nerve distribution was transient in all patients.

**Conclusion:**

Reverse sural artery flap remains to be the workhorse flap to resurface the soft tissue defects of the foot and ankle. Anastomosis of the sural nerve to the digital plantar nerve can potentially solve the issue of lack of sensation in the flap especially when used for weight bearing heel.

## Background

Soft tissue defects around the foot and ankle region often present an awkward problem to the orthopaedic surgeons. Tendons and bones are frequently exposed after trauma because of the thinness of the subcutaneous tissue. Traditionally, local flaps were used for resurfacing but their use is limited by their size and arc of rotation. Reverse flow flaps such as anterior tibial artery flap and posterior tibial artery flap require the sacrifice of major arteries. Free flap is currently the treatment of choice for large soft tissue defects of the distal extremity and it solves the problem of donor site morbidity in the immediate vicinity of the flap. It is however a technically demanding procedure for surgeons with less microsurgical experience. Furthermore in a few cases of trauma with damaged or occluded major vessels, where a free flap may be potentially hazardous, the reverse sural artery flap can prove to be one of the few safe options for soft tissue coverage [1]. The accompanying arteries of the lesser saphenous vein and sural nerve have been utilized with success for harvest of reverse flow sural flap [2]. The sural nerve remains the anatomic landmark for the inclusion of vessels in pedicle of the flap. The sural artery reverse flow flap is nourished by the lowermost perforating branch of the peroneal artery. Since the introduction of this flap by Masquelet [3] in 1992, it has become one of the favourite among reconstructive surgeons. It is the flap of first choice in our centre. In the present report, the results of reverse flow sural neurocutaneous flaps done at our centre is presented along with some thought provoking ideas for the future.

## Methods

This descriptive case series was conducted between 1997 and 2003, at Queen Mary Hospital, The University of Hong Kong, Division of Hand Surgery, Department of Orthopaedics and Traumatology. It is a tertiary care centre for cases requiring microsurgery of the extremities. From 1997 to 2003, we performed ten reverse sural neurocutaneous flaps on ten patients (See Table 1). The age ranged from 19 to 85 years, with a mean age of 59.8 years. There were six females (60%) and four male patients (40%). The soft tissue defects were located on non weight bearing heel in four patients (40%), tendo Achilles in two patients (20%), distal tibia in two patients (20%), lateral malleolus in one patient (10%) and medial aspect of the midfoot in one patient (10%). No patient underwent resurfacing of the weight bearing heel. The minimum and maximum flap size harvested (length × breadth in cm.) was 5 × 4 and 14 × 6 respectively. A midline cuff of gastrocnemius was included in one of the flap harvested from the proximal calf area. All benefits and disadvantages of the operation were discussed with the patient before the operation, including the sensory loss in the distribution of the sural nerve. A detailed questionnaire was developed using variables of interest.

**Table 1:** Patients' data. Demographic features, etiology, defect site and size, comorbids, flap type and outcome

**Sex**	**Age**	**Etiology**	**Site of defect**	**Flap size (cm)**	**Comorbids**	**Flap type**	**Complications**
F	72	Ulcer	Medial aspect of mid foot	9 × 7	DM	Sural	Mild venous congestion
M	74	Pressure sore	Lateral malleolus	5 × 4	Paraplegia	Sural	
F	68	Ulcer	NWB heel	8 × 6	DM	Sural	
F	62	Trauma	NWB heel and medial malleolus	11 × 7		Sural	
M	48	Trauma	Tendoachilles	8 × 6		Sural	
F	53	Trauma	Shin	8 × 6		Sural	
F	19	Trauma	NWB heel	14 × 6		Sural	
M	52	Trauma	Distal tibia	9 × 7		Sural	
F	85	Ulcer	Achilles tendon	7 × 4	PVD, DM	Sural	
M	65	Ulcer	Posterior heel	8 × 5	DM	Sural	

Preoperative evaluation included identification of the site of peroneal perforators above the lateral malleolus using Doppler flow meter. Two or three perforators were identified above the lateral malleolus. The pivot point of the pedicle was chosen according to the need of distal coverage, but it was limited by the lower most perforator, about 05 cm. from the tip of the lateral malleolus.

**Figure 1:**
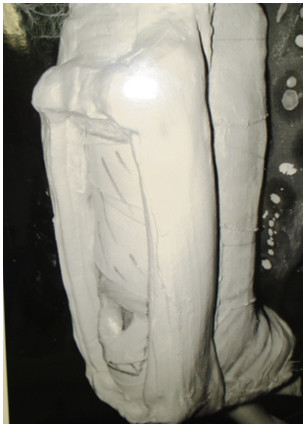
**POP boot**. A 'modified plaster of paris boot' is designed to make sure that there is no pressure on the flap pedicle even if the patient is lying supine in bed. One can appreciate the built in walls of the boot on the posterior aspect, with the flap visible from within them.

The skin was incised close to the midline, so as to be sure to include the short saphenous vein in the pedicle in all patients. The skin flap was then elevated with the deep fascia. The pedicle was kept wide, around 03 to 04 cm. in diameter. The flap was rotated 180 degrees through an open tunnel in all but one patient. Split thickness skin graft was used to cover the exposed pedicle in all but one patient. All patients were kept in the 'modified plaster of paris boot' in the post operative period (Figure 1).

## Results (Also see Table 1)

### Survival

All flaps survived.

### Venous congestion

Only one flap developed mild venous congestion in the early post operative period, which resolved with foot elevation.

### Weight bearing problems

None of our patients had resurfacing to the weight bearing part of the heel. There was no interference with walking.

### Donor and recipient site appearance

The donor site had no complications except for the relatively unsightly scar. It was acceptable to all patients. All patients were satisfied with the cosmesis of the recipient site.

### Numbness/Neuroma

Transient numbness along the sural nerve distribution was experienced by the patients. It resolved in all patients by three months. No painful neuroma developed as we routinely buried the nerve stump in the deep muscular plane.

### Need for debulking of the flap

No patients required debulking for cosmetic reasons or shoe wear problems.

## Case reports

### Case report 1 (Figures 2A and 2B)

**Figure 2:**
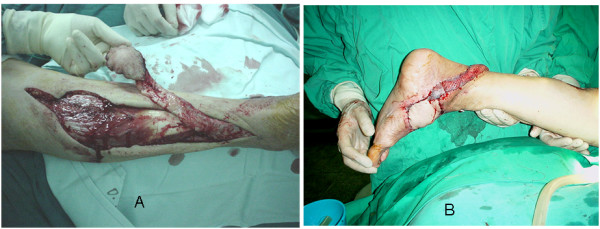
**A – CR. 1, flap elevation**. 72 years lady was suffering from ulcer on dorso medial aspect of midfoot as a result of long standing diabetes mellitus leading to peripheral neuropathy. Sural artery flap was utilized; its elevation is seen from the proximal aspect of the posterior calf area, modified by the inclusion of midline gastrocnemius muscle cuff around the sural pedicle. **B – CR. 1, Post op**. Adequate coverage seen in the immediate post operative period. The pedicle was kept wide and not passed through subcutaneous tunnel. It required split thickness skin grafting for coverage. The flap developed mild distal congestion which resolved spontaneously with foot elevation without any problems.

72 years diabetic lady suffering from ulcer on the dorsomedial aspect of the right midfoot was successfully covered using the reverse sural flap from proximal calf area with the modification of inclusion of the midline cuff of gastrocnemius, as described by Al Qattan MM [4].

### Case report 2 (Figures 3A, 3B, 4A, 4B and 4C)

**Figure 3:**
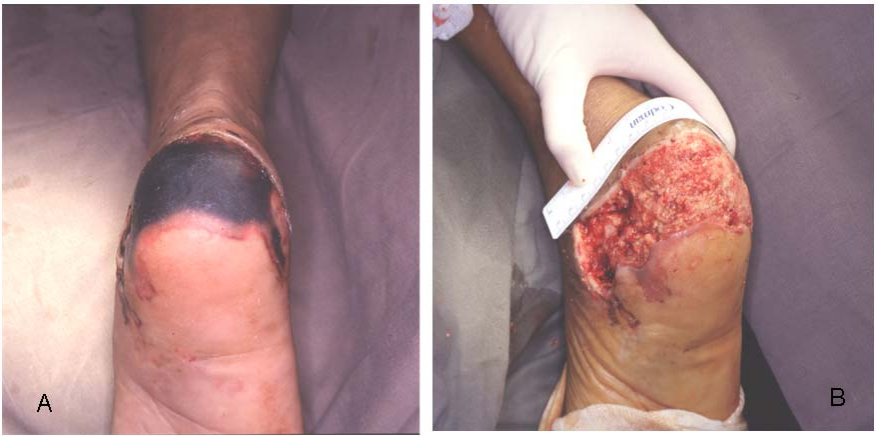
**A – CR. 2, pre op**. 19 years lady suffered road traffic accident resulting in skin necrosis of the posterior aspect of the heel. **B – CR. 2, post debrima**. After debridement, area of skin loss can be seen, optimal for flap coverage.

**Figure 4:**
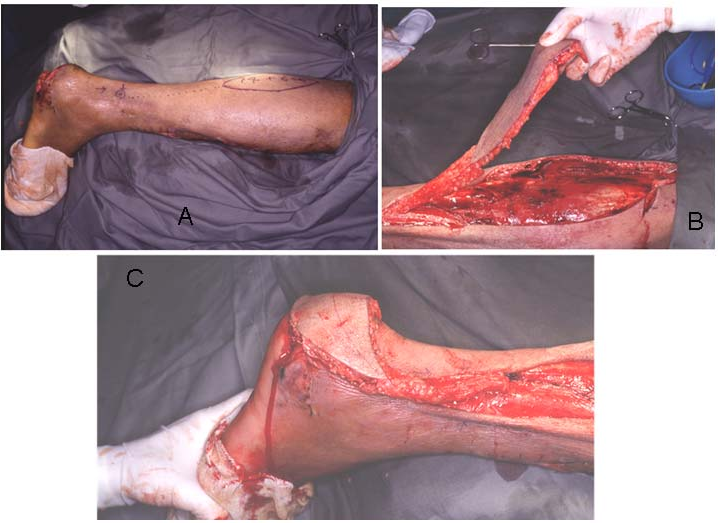
**A – CR. 2, flap marking**. The sural artery neurocutaneous flap is marked on the skin with dimensions, 14 × 6 cm. **B – CR. 2 – flap rotation**. The reverse flow sural artery neurocutaneous flap is being rotaed through an arc 180 degrees on its pedicle. **C – CR. 2 – outcome**. The flap inset into the defect after rotation with excellent coverage.

19 years lady sustained road traffic accident resulting in skin necrosis of the posterior aspect of the left heel. Reverse sural flap size 14 × 6 cm. was used to successfully cover the defect.

## Discussion

The results of this study show that the reverse sural neurocutaneous flap is an effective method to resurface soft tissue defects around the foot and ankle region.

We would recommend some measures that we adopted in our cases and which have been advised in the literature to ensure safe flap elevation and improve the venous congestion of the flap, hence decrease the risk of failure.

1) Preoperative identification of the peroneal artery perforators by Doppler should be performed and the peroperative search for a perforator should be avoided which can be potentially dangerous [1].

2) A 'modified plaster of paris boot' (Figure 1), that we describe in our cases can be used to avoid pressure on the pedicle in the postoperative period. This helps the patients to remain supine, allows assessment of the flap circulation and assists in the elevation of the foot to relieve venous congestion.

3) The pedicle should be kept wide (3 to 4 cm.) [1,3] and skin grafting should be done on the pedicle if the subcutaneous tunnel becomes tight and impairs circulation. In our series, in nine out of ten patients the pedicle was passed through an open tunnel and skin grafted. Although skin grafting added slightly to the donor site morbidity, it prevented pressure on the pedicle in the post operative period.

4) The lesser saphenous vein should be included in the pedicle in all cases [2].

5) There are a few studies in which this flap is used to cover defects in the distal part of the foot by slight modification of the flap design, by inclusion of the midline cuff of gastrocnemius muscle, in flaps harvested from proximal calf area [4]. We performed one flap in this manner to cover the medial aspect of the midfoot in an old lady with diabetic ulcer (Figure 2A and 2B). We postulate that the whole skin of the posterior calf can be harvested without any major problem. Sural neurofasciocutaneous flaps as large as 17 × 16 cm. have been reported [5].

6) Leech therapy can also be used to decrease venous congestion [6]. We did not require the use of leech therapy in cases of reverse sural neurocutaneous flaps but did found it of use in a reverse flow saphenous neurocutaneous flap.

Anastomosis with one of the donor veins from the foot may also be an alternative, but we do not see the need for this intervention at the moment.

The vascular anatomy and clinical application of the reverse sural neurocutaneous flap has been well studied [2,7-10]. The artery existed as an axial pattern vessel in only 50 percent of our patients.

Self limiting numbness in the distribution of sural nerve is not a major concern. The patients should be counselled preoperatively and the problem is usually resolved on an average in three months in all patients. To avoid a painful neuroma, the nerve stump needs to be buried in the deep muscular plane.

Donor site morbidity was minimal in our patients. Unaesthetic donor site, perhaps, may be a major concern to the young female patients. It can potentially be avoided by using an adipofascial flap, rather than harvesting skin island in larger flaps.

### Future Concern

Based on the sensate character and same quality skin, medial plantar flap has been proposed to be superior for heel coverage over sural artery reverse flow flaps [11,12]. However no substantial clinical or laboratory data is available for reinnervating the reverse sural artery flaps. Reverse flow homodigital island flaps for finger tip injuries have been rendered sensate by including a segment of palmar digital nerve in the flap design and anastomosing it to the opposite side digital nerve [13-15].

Based on the above observations we postulate end to side anastomosis of the sural nerve to a plantar digital nerve, in order to provide sensory reinnervation to the flap especially in cases of weight bearing heel reconstruction.

## Conclusion

We highly recommend this flap to resurface soft tissue defects in the foot and ankle region because it is easy to harvest, reliable and can be used in patients with peripheral vessel disease and trauma patients with damaged major vessels [1].

## List of abbreviations

POP: plaster of paris, CR: case report, pre op: pre operative, post op: post operative, debrima: debridement, NWB: non weight bearing, F: female, M: male, PVD: peripheral vascular disease, DM: diabetes mellitus, cm: centimetres

## Competing interests

The authors declare that they have no competing interests.

## Authors' contributions

SKA carried out patient follow up, concept design, data collection, literature search, manuscript writing and critical revision. BKKF carried out surgery, patient follow up, concept design, manuscript writing and literature search. WYI carried out surgery, patient follow up, literature search. MF carried out patient follow up, data collection. SPC carried out surgery, review and final approval of the manuscript. All authors read and approved the final manuscript.
